# Supplementary material for: Disentangling metabolic functions of bacteria in the honey bee gut
Source: PLoS Biol. 2017 Dec 12;15(12):e2003467. doi: 10.1371/journal.pbio.2003467 (PMC5726620; doi:10.1371/journal.pbio.2003467)
Supplement: S8 Data — (ZIP) [file pbio.2003467.s008.zip › Z-score_plots/820 microbial substrate Glenvastatin.pdf]

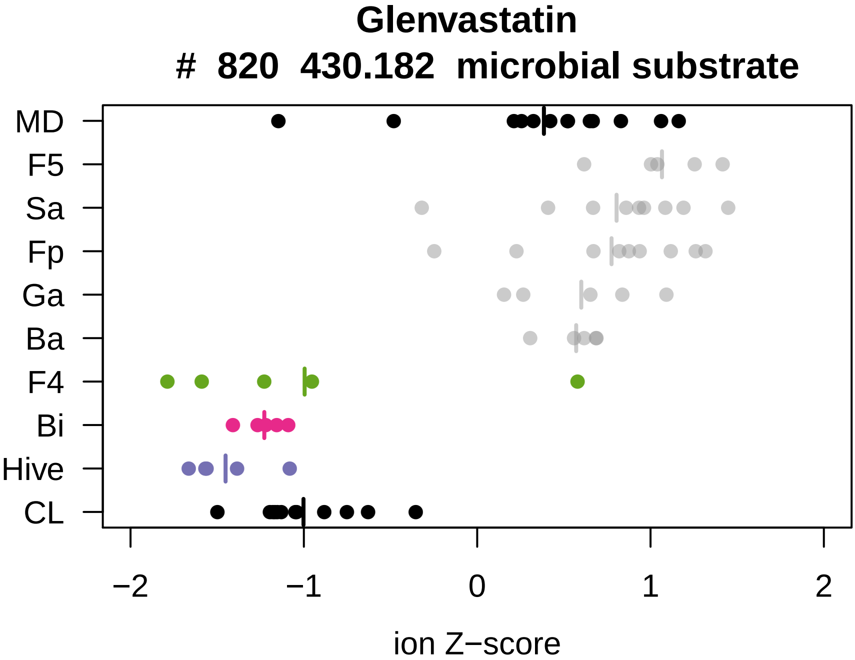
Glenvastatin
820 430.182 microbial substrate
MD
F5
Sa
Fp
Ga
Ba
F4
Bi
Hive
CL
−2
−1
0
1
2
ion Z−score
